# Supplementary material for: Risk Assessment of the Dietary Phosphate Exposure in Taiwan Population Using a Total Diet Study
Source: Foods. 2020 Oct 30;9(11):1574. doi: 10.3390/foods9111574 (PMC7692739; doi:10.3390/foods9111574)
Supplement: Supplementary file 1 [file foods-09-01574-s001.pdf]

Appendix-Concentrations of total phosphorus detected in the 168 food samples(mg/kg or mg/L as appropriate)

Food categories	Food groups	Analyte concentrati
		Phosphorus
01 Dairy products and analogues	01.1.1 Milk and butter milk-UHT milk	348.8
	01.1.1 Milk and butter milk-Milk	1228.2
	01.1.2 Dairy-based drinks, flavoured and/or fermented	431.2
	01.2.1 Fermented milks	746.1
	01.3.1 Condensed milk	1525.5
	01.3.2 Beverage whitener-Liquid	912.7
	01.3.2 Beverage whitener-Powder	921.1
	01.3.2 Beverage whitener-Powder	5281.1
	01.3.2 Beverage whitener-Powder	4664.5
	01.3.2 Beverage whitener-Liquid	1447.0
	01.4 Cream and the like	719.0
	01.5.1 Milk powder and cream powder-Skimmed milk powder	11056.4
	01.5.1 Milk powder and cream powder-Growth milk powder	4999.9
	01.5.1 Milk powder and cream powder-Milk powder	7793.3
	01.5.2 Milk and cream powder analogues	5049.0
	01.6 Cheese and analogues-Cheese analogues	8467.2
	01.6 Cheese and analogues-Unripened cheese	3051.7
	01.7 Dairy-based desserts-Pudding	548.2
	01.7 Dairy-based desserts-Yoghurt	920.5
	01.8 Whey and whey products-Dried whey and whey products	2279.7
02 Fats and oils, fat emulsions	02.1.2 Vegetable oils and fats	Non-detected
	02.1.3 Lard, tallow, fish oil, and other animal fats	Non-detected
	02.2.1 Butter	39.1
	02.2.2 Fat spreads, dairy fat spreads and blended spreads	64.5
	02.3 Fat emulsions mainly of type oil-in-water, including mixed and/or flavoured products based on fat emulsions	283.5
03 Edible ices	03.1 Ice cream	879.3
	03.2 Popsicle	593.6
	03.3 Edible ices	Non-detected
	03.4 Other ices	126.0
	04.1.1 Fresh fruit	132.6
	04.1.2.2 Dried fruit	788.7
	04.1.2.2 Dried fruit	965.7
	04.1.2.6 Fruit-based spreads (e.g. chutney) excluding products of food category 04.1.2.5	177.7
	04.1.2.7 Candied fruit	327.7
	04.1.2.8 Fruit preparations, including pulp, purees, fruit toppings and coconut milk	71.5
	04.1.2.9 Fruit-based desserts, incl. fruit-flavoured water-based desserts	100.2
	04.1.2.11 Fruit fillings for pastries	28.6
	04.2.1.1 Untreated fresh vegetables	372.0
	04.2.1.2 Surface-treated fresh vegetables	455.4
	04.2.1.3 Peeled, cut or shredded fresh vegetables	258.0

		**Phosphorus**
04 Fruits and vegetables	04.2.2.1 Frozen vegetables	664.3
	04.2.2.2 Dried vegetables-Dried seaweeds	419.8
	04.2.2.2 Dried vegetables-Dried mushrooms	914.2
	04.2.2.3 Vegetables in vinegar, oil, brine, or soybean sauce	292.8
	04.2.2.3 Vegetables in vinegar, oil, brine, or soybean sauce-Dried bamboo	436.3
	04.2.2.4 Canned or bottled (pasteurized) or retort pouch vegetables	591.6
	04.2.2.5 Vegetable purees and spreads	1066.8
	04.2.2.6 Vegetable pulps and preparations	855.7
	04.2.2.7 Fermented vegetable products	180.8
	04.2.2.8 Cooked or fried vegetables	714.7
	04.2.2.9 Cooked beans and bean paste products	680.3
	04.2.2.10.2 Soybean-based beverages	190.8
	04.2.2.10.3 Tofu skin products	2155.0
	04.2.2.10.4 Soybean curd -Tofu	1727.0
	04.2.2.10.4 Soybean curd -Oily tofu	2078.7
	04.2.2.10.5 Dehydrated soybean curd (kori tofu)	2930.0
	04.2.2.10.6 Fermented soybeans (e.g. natto, tempe)	1832.9
05 Confectionery	05.1.1 Cocoa mixes (powders) and cocoa mass/cake	5241.1
	05.1.3 Cocoa-based spreads, incl. fillings	988.4
	05.1.4 Cocoa and chocolate products	3179.4
	05.1.5 Imitation chocolate, chocolate substitute products	2052.5
	05.2.1 Hard candy	74.2
	05.2.2 Soft candy	53.7
	05.2.3 Nougats and marzipans-Nougats	1647.7
	05.2.3 Nougats and marzipans-Marzipans	2151.6
	05.3 Chewing gum	149.2
	05.4 Decorations, toppings (non-fruit), and sweet sauces	1641.0
06 Cereals and cereal products	06.1 Whole, broken, or flaked grain, including rice	1381.3
	06.2 Roots, tubers and other non-cereal crops	633.1
	06.3.1.1 Flours	943.8
	06.3.1.2 Rice flours	1330.4
	06.3.1.3 Other cereals, roots and tubers flour products	4410.7
	06.3.2 Starches	138.0
	06.4.1 Fresh pastas and noodles	181.3
	06.4.1 Fresh pastas and noodles-Only noodles	251.0
	06.4.2 Dried pastas and noodles	228.7
	06.4.2 Dried pastas and noodles-Only noodles	240.1
	06.4.3 Pre-cooked pastas and noodles	1048.8
	06.5 Puffed food with flour or starch as raw material-Vegetable buns	455.6
	06.5 Puffed food with flour or starch as raw material	653.2
	06.5 Puffed food with flour or starch as raw material-Meat buns	777.2
	06.6 Breakfast cereals	3335.4
	06.7 Cereal and starch based desserts-Tapioca balls	27.3
	06.7 Cereal and starch based desserts	210.7
	06.9 Batters	2349.4
	06.11 Pre-cooked or processed rice products	1130.3
	07.1.1.1 Yeast-leavened breads and specialty breads	769.0

		Phosphorus
07 Bakery wares	07.1.1.2 Soda breads	668.7
	07.1.2 Crackers, excluding sweet crackers	1019.9
	07.1.3 Other ordinary bakery products (e.g. bagels, pita, English muffins)	960.4
	07.1.4 Bread-type products, including bread stuffing and bread crumbs	757.3
	07.1.5 Steamed breads and buns	1104.9
	07.2.1 Cakes, cookies and pies (e.g. fruit-filled or custard types)	1764.5
	07.2.2 Other fine bakery products (e.g. doughnuts, sweet rolls, scones, and muffins)	597.7
	07.2.2 Other fine bakery products (e.g. doughnuts, sweet rolls, scones, and muffins)	924.2
	07.2.3 Mixes for fine bakery wares	2584.2
08 Meat and meat products	08.1 Fresh meat, poultry, and game-Beef	1409.1
	08.1 Fresh meat, poultry, and game-Chicken	1654.6
	08.1 Fresh meat, poultry, and game-Pork	1762.6
	08.2.1.1 Cured (including salted) non-heat treated processed meat, poultry, and game products in whole pieces or cuts	1549.6
	08.2.1.2 Cured (including salted) and dried non-heat treated processed meat, poultry, and game products in whole pieces or cuts	1373.2
	08.2.2 Heat-treated processed meat, poultry, and game products in whole pieces or cuts	1799.7
	08.2.3 Frozen processed meat, poultry and game products in whole pieces or cuts	1258.3
	08.3.1 Non-heat treated processed comminuted meat, poultry, and game products	1929.1
	08.3.2 Heat-treated processed comminuted meat, poultry, and game products	989.0
	08.3.3 Frozen processed comminuted meat, poultry, and game products	1873.4
	08.4.4 Ham (smoked, smoked, steamed)	1950.6
	08.4.7 Cooked meat jerky	1733.4
	08.4.8 Canned meat	1152.1
	08.4.9 Edible casings (e.g. sausage casings)	220.2
09 Fish and fish products	09.1.1 Fresh fish	2027.1
	09.1.2 Fresh molluscs, crustaceans, and echinoderms-Squid	1586.6
	09.1.2 Fresh molluscs, crustaceans, and echinoderms-Clams	1838.8
	09.1.2 Fresh molluscs, crustaceans, and echinoderms-Shrimp	2375.1
	09.2.1 Frozen aquatic products	1159.0
	09.2.2 Cooked and/or fried aquatic products	2182.4
	09.2.3 Smoked, dried, fermented, and/or salted aquatic products-Dried shrimp	6287.0
	09.2.3 Smoked, dried, fermented, and/or salted aquatic products-Dried fish	12552.0
	09.2.4 Surimi	1203.7
	09.2.6 Aquatic products pickled and/or in brine	2145.5
	09.2.7 Canned aquatic products	2040.1
	09.4 Cooked seafood aquatic products	3830.3
10 Egg and egg products	10.1 Fresh eggs	2938.0
	10.2.1 Liquid egg products	105.3
	10.2.3 Dried and/or heat coagulated egg products	1866.9
	10.3 Preserved eggs, including alkaline, salted, and canned eggs	1828.9
	10.4 Egg-based desserts (e.g. custard)	364.5
	11.1.1 White sugar, dextrose anhydrous, dextrose monohydrate, fructose	Non-detected

		Phosphorus
11 Sweeteners, including honey	11.1.2 Powdered sugar, powdered dextrose	1.8
	11.2 Brown sugar	138.3
	11.4 Other sugars and syrups	Non-detected
	11.6 Table-top sweeteners, including those containing high-intensity sweeteners	28.4
12 Salts, spices, soups, salads, and protein products	12.1.1 Salt-Refined salt	Non-detected
	12.1.1 Salt-Rock salt	Non-detected
	12.1.2 Salt substitutes	465.8
	12.2.1 Herbs and spices	1009.9
	12.2.2 Seasonings and condiments-meat tenderizer	48.1
	12.2.2 Seasonings and condiments-Curry cubes	469.3
	12.2.2 Seasonings and condiments-MSG	803.9
	12.2.2 Seasonings and condiments-Other seasonings	1207.5
	12.3 Vinegars	30.0
	12.5.1 Ready-to-eat soups and broths-Canned sweet soups	380.3
	12.5.1 Ready-to-eat soups and broths-Broth	49.7
	12.5.1 Ready-to-eat soups and broths-Thick soup	337.7
	12.5.2 Mixes for soups and broths	985.0
	12.6.1 Emulsified sauces and dips	225.2
	12.6.2 Non-emulsified sauces	409.1
	12.6.3 Mixes for sauces and gravies	1074.0
	12.6.4 Clear sauces (e.g. fish sauce)	313.9
	12.9.1 Fermented soybean paste (e.g. miso)	1512.8
	12.9.2 Soybean sauce	1257.6
13 Foodstuffs intended for particular nutritional uses	13.2 Complementary foods for infants and young children	3520.7
	13.3 Dietetic foods intended for special medical purposes	622.4
	13.4 Dietetic formulae for slimming purposes and weight reduction	7110.2
	13.5 Other dietetic foods	8811.3
14 Beverage	14.2.1.1 Fruit juice	92.3
	14.2.1.3 Concentrates for fruit juice	187.8
	14.2.2.1 Fruit nectar	7.7
	14.2.2.2 Vegetable nectar	98.3
	14.2.3.1 Carbonated water-based flavoured drinks	81.2
	14.2.3.2 Non-carbonated water-based flavoured drinks-Teas	11.1
	14.2.3.2 Non-carbonated water-based flavoured drinks-Milk teas	92.2
	14.2.3.3 Concentrates (liquid or solid) for water-based flavoured drinks	116.5
	14.2.4 Coffee, coffee substitutes, tea, herbal infusions, and other hot cereal and grain beverages, excluding cocoa	25.8
15 Processed snacks and nut products	15.1 Snacks - potato, flour or starch based (from roots and tubers, pulses	1489.0
	15.2 Processed nuts-Peanuts	4322.1
	15.2 Processed nuts-Melon seeds	9039.7
16 Dietary supplements	16.0 Food supplements	774.3

		Phosphorus
